# Alport Syndrome May contribute to Grand Multiparity in a Typical Low Income Setting

**DOI:** 10.4314/ejhs.v35i1.10

**Published:** 2025-01

**Authors:** Victoria I Ogala-Akogwu, Peter K Uduagbamen, Emmauuel A Anteyi, Habib A Galadanci

**Affiliations:** 1 Nephrology Unit, Department of Medicine, State House Medical Centre, Abuja, Nigeria; 2 Nephrology Unit, Department of Medicine, National Hospital, Abuja, Nigeria; 3 Division of Nephrology and Hypertension, Department of Internal Medicine, Bowen University/Bowen University Teaching Hospital, Ogbomosho, Nigeria

**Keywords:** Alport syndrome, hemodialysis, kidney transplant, X-linked, hematuria, mutation, collagen IV, end-stage kidney disease

## Abstract

Alport syndrome (AS) is a rare, inherited disorder affecting the basement membranes of the glomerulus, cochlea, and lens. It presents with visual and hearing deficits, as well as kidney disease, which can progress to end-stage renal failure and death. A 26-year-old male presented with a three-week history of body swelling, foamy urine, worsening hematuria, and hearing and visual impairments of 17- and 10-years' duration, respectively. Three of his siblings (two males and one female) had similar symptoms. Molecular genetic screening, involving children from the two wives, identified a pathogenic mutation in the COL4A5 gene, confirming X-linked AS in the patient and his three maternal siblings. The patient underwent maintenance hemodialysis (HD), followed by two failed living-related kidney transplants, and died in his sleep a year after the second transplant, hours after a routine dialysis session.

Multiple deaths from AS within a family can contribute to grand multiparity, particularly in low-income settings.

## Introduction

Alport syndrome (AS), first described in 1927 by Cecil Alport, is a rare monogenic disorder that affects the basement membranes (BM) of the glomerulus, cochlea, lens, and skin (1). Molecular cloning and sequencing, which identified the collagen isoforms COL4A1 and COL4A2 in 1987, later revealed six isoforms of collagen (α1(IV) to α6(IV)) along with their respective protein domains (2). A multiracial, multiethnic study assessing the global prevalence of predicted pathogenic variants (PPV) in AS found an overall prevalence of 1 in 2320. However, this varies by region, with PPV in COL4A5 being most common in Africans (1 in 1733), absent in Finns, and rare in Ashkenazi Jews (1 in 4961). The PPVs for COL4A3 and COL4A4 are 0.41% and 0.42%, respectively (3). Variants in COL4A3 and COL4A4 are most prevalent in Latinos (1.58%), East Asians (1.34%), and Africans (1.28%). These findings suggest that, despite limited tissue biopsy and molecular genetic testing in resource-limited settings, the prevalence of Alport syndrome in Africa is likely high (4).

Hematuria in AS is often preceded by visual and hearing impairments (5). Hematuria and proteinuria are associated with high-tone sensorineural deafness and anterior lenticonus (1-2, 5). Here, we present the case of an African renal graft recipient secondary to AS and explore its association with grand multiparity.

## Case Review

A 26-year-old male undergraduate was referred for evaluation of a three-week history of body swelling, foamy urine, worsening hematuria, myopia, and hearing loss, diagnosed at ages 9 and 16, respectively. He had no personal or family history of hypertension or diabetes and was the fourth of eight children in a polygamous family. Three siblings (two males and one female) also exhibited similar symptoms.

Physical examination revealed facial puffiness and pallor. His blood pressure was 150/90 mmHg, and he had asterixis. The diagnosis of end-stage kidney disease (ESKD) secondary to chronic glomerulonephritis (CGN) was made. His renal biochemistry (Table 1) prompted the initiation of the first hemodialysis (HD) session. Urinalysis showed proteinuria (1+) and hematuria (2+). Chest X-ray and electrocardiography (ECG) were normal. A renal scan revealed shrunken kidneys with poor cortico-medullary differentiation. Serological tests ruled out hepatitis B and C.

**Table 1:** Laboratory results at presentation

Sodium in mmol/L	Potassium in mmol/L	Urea in mmol/L	HCO_3_ in mmol/L	SCr in µmol/L	eGFR in mL/min	TP in g/dL	Albumin in g/dL	CCal in mmol/L
(135-145)	(3.5-5.0)	(3-7)	22-30	(70-120)	≥90	(60-80)	(35-50)	(2.2-2.5)

135	5.8	38.5	16	928	7.3	62	28	2.10
	
WBC x 109/L	HC g/dL	PCV %	MCV fl	Platelets/mm^3^	Diff-N %	Diff-L %	ESR mm/hr	
4-11	13-17	40-52	76-96	150-400	45-65	25-45	<7	
	
6.4	9.6	30	74	259	65	33	17	
					
T. chol mmol/L	HDL-C mmol/L	LDL-C mmol/L	Trig mmol/L					
3.5-5.2	>1.1	<3.0	<1.7					
					
3.2	0.7	2.2	0.4					

HCO3-bicarbonate, Crc-creatinine, eGFR-estimated glomerular filtration rate, TP-total protein, CCal-corrected calcium, WCC-white cell count, HC-haemoglobin concentration, PCV-packed cell volume, MCV-mean corpuscular volume, ESR-erythrocyte sedimentation rate, HDLC-high density lipoprotein, LDLC-low density lipoprotein cholesterol

Genetic counseling was offered due to similar symptoms in maternal siblings, and molecular genetic testing (performed in India) confirmed a COL4A5 mutation in the patient and three maternal siblings, establishing the diagnosis of X-linked Alport syndrome.

Following the first HD session, the patient was managed conservatively for three months before initiating maintenance hemodialysis (MHD) twice weekly. He underwent a living-related kidney transplant (LRKT) from his 40-year-old paternal aunt. His allograft function remained stable for 18 months, after which renal function deteriorated, leading to graft failure. He resumed MHD and was worked up for a second LRKT five years later, this time from his 42-year-old paternal aunt. However, the transplant failed on postoperative day 3, and he returned to thrice-weekly MHD.

Due to the stress of managing school and dialysis, he opted to leave university. He remained stable on MHD for two years until he died suddenly in his sleep, a day after his routine dialysis session. Neither the patient nor his sibling underwent a kidney biopsy.

## Discussion

Alport syndrome (AS), also known as hereditary nephritis, is a genetically and phenotypically heterogeneous disease caused by mutations in genes encoding type IV collagen, specifically the alpha-3, alpha-4, and alpha-5 chains (COL4A3, COL4A4, COL4A5) (6). The classical syndromic pattern is seen in the typical patient, who presents with bilateral sensorineural deafness, myopia, and progressive kidney disease leading to end-stage renal disease (ESRD).

AS is more common in males, who rend to exhibit more severe symptoms (1, 5). The death of the only affected female highlights subsequent findings of other AS variants, including autosomal recessive and autosomal dominant forms, with the latter being less severe (7). The “Expert Guidelines for the Diagnosis and Management of Alport Syndrome” recommend that women with AS be monitored annually for albuminuria, as the presence of this may indicate severe disease. If present, management should include renin-angiotensin-aldosterone system (RAAS) inhibition alongside a birth control program (8).

X-linked AS typically presents earlier and is associated with more severe symptoms and worse outcomes, as observed in the index patient and his three siblings, all of whom died from the disease, including the female with heterozygous X-linked AS. Hematuria, the most common initial symptom of AS, often follows ocular and visual deficits. 5 Typical ocular manifestations include dot-and-fieck retinopathy (85%), anterior lenticonus (25%), and, rarely, posterior polymorphous corneal dystrophy (9). The penetrance of hematuria in COL4A5 is high in both males and females, especially in males with kidney disease. Persistent hematuria is also common in COL4A3/COL4A4 variants, with a prevalence of approximately 70%. The high prevalence of AS among Africans, particularly in those with COL4A5 mutation and to a lesser extent COL4A3 and COL4A4 mutations, confirms its significant impact in this population (3,4).

The presence or absence of alpha-3, -4, and -5 collagen chains in the glomerular basement membrane (GBM) can be assessed using monoclonal antibodies, with absence confirming the disease (7, 8). Light microscopy (LM) findings may be normal in early stages, while immunofluorescence (IF) staining may show no signal. Electron microscopy (EM) in early stages shows GBM thinning and splitting, while late stages may reveal focal segmental glomerulosclerosis (FSGS), tubular atrophy, interstitial fibrosis, and lymphocytic and plasma cell infiltration (7, 8).

The pathophysiology of AS involves abnormal production or assembly of type IV collagen fibers, leading to dysfunctional renal filtration and subsequent basement membrane fibrosis and scarring (8). Recent guidelines have recommended removing the term “carriers,” as individuals may develop thin basement membrane nephropathy (TBMN), which can progress to ESRD. Qazi et al reported that 7.4% of all kidney biopsies had TBMN, a high prevalence that may impact the availability of living-related kidney donors, the primary option in many regions (10). This highlights the case of the index patient, who could only receive a renal graft from his paternal family. Ongoing trials are exploring various agents, including sodium/glucose cotransporter-2 inhibitors (SGLT2is), endothelin type A antagonists, and gene replacement therapy (2, 6, 10).

Grand multiparity remains a significant cultural factor in many African and low socioeconomic communities, driven by the fear of multiple child deaths from epidemics and other causes. Women, particularly, desire many children, believing that even with several deaths, some will survive (11). The fact that the index mother had four children after losing four underscores this belief and may encourage younger women to embrace grand multiparity as a way to prepare for life's uncertainties. In 1988, the Nigerian government implemented a policy limiting the number of children per couple to four, down from an average of five to six (12). This policy is also reflected in the National Health Insurance Scheme (NHIS), which restricts the number of enrollees per couple to four (13). The index patient and his late siblings were born after this policy was enacted. Their father, a senior public servant, was able to afford genetic testing for all his children, an investigation not covered by NHIS, demonstrating the persistence of grand multiparity in this population.

The index patient's sudden death in his sleep, a day after hemodialysis (HD), aligns with findings that HD patients are at an increased risk of sudden death within the first 12 hours post dialysis, as well as during intradialytic and 48-60 hours post dialysis periods. This is especially common in those on the Monday-Wednesday-Friday schedule (or Tuesday-Thursday-Saturday), with arrhythmia-associated cardiovascular disease (AACVD) often implicated (14). Disease progression can worsen left ventricular hypertrophy (LVH) and increase the risk of myocardial ischemia, hibernation, stunning, and AACVD. Channelopathies such as Brugada syndrome (12%) and those related to serum potassium levels, which are common causes of sudden cardiac death (SCD), were largely ruled out through periodic ECGs (14).

Management of Alport syndrome requires a multidisciplinary approach, involving nephrologists, geneticists, genetic counselors, audiologists, ophthalmologists, transplant surgeons, nutritionists, and social workers, among others (7, 10). Hyperacute rejection from anti-GBM disease should be considered, as preemptive plasmapheresis may minimize this complication. Kidney transplantation in AS is generally favorable due to the lack of disease recurrence; however, 5% of transplant cases may develop anti-glomerular basement membrane disease (Anti-GBM), often with higher graft failure rates (15).

Early onset hematuria in children, with or without ocular and visual deficits, should raise suspicion for AS and prompt further investigation. Management should involve a multidisciplinary team. The desire for multiple children due to fears of losing them to pandemics and rare diseases may drive grand multiparity, particularly in traditional African settings.

## Figures and Tables

**Figure 1:**
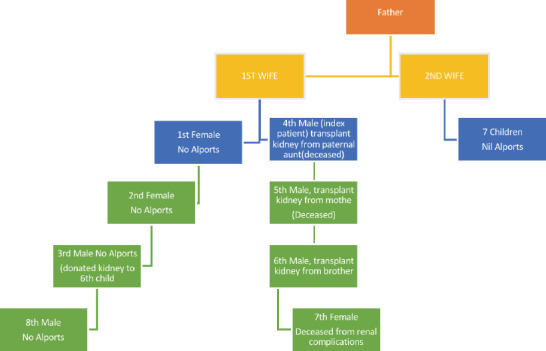
Family tree of the index patient
